# The early bird catches the term: combining twitter and news data for event detection and situational awareness

**DOI:** 10.1186/s13326-016-0103-z

**Published:** 2016-10-07

**Authors:** Nicholas Thapen, Donal Simmie, Chris Hankin

**Affiliations:** Institute for Security Science and Technology, Imperial College London, Exhibition Road, London, UK

**Keywords:** Twitter, Situational awareness, Event detection

## Abstract

**Background:**

Twitter updates now represent an enormous stream of information originating from a wide variety of formal and informal sources, much of which is relevant to real-world events. They can therefore be highly useful for event detection and situational awareness applications.

**Results:**

In this paper we apply customised filtering techniques to existing bio-surveillance algorithms to detect localised spikes in Twitter activity, showing that these correspond to real events with a high level of confidence. We then develop a methodology to automatically summarise these events, both by providing the tweets which best describe the event and by linking to highly relevant news articles. This news linkage is accomplished by identifying terms occurring more frequently in the event tweets than in a baseline of activity for the area concerned, and using these to search for news. We apply our methods to outbreaks of illness and events strongly affecting sentiment and are able to detect events verifiable by third party sources and produce high quality summaries.

**Conclusions:**

This study demonstrates linking event detection from Twitter with relevant online news to provide situational awareness. This builds on the existing studies that focus on Twitter alone, showing that integrating information from multiple online sources can produce useful analysis.

## Introduction

Updates posted on social media platforms such as Twitter contain a great deal of information about events in the physical world, with the majority of topics discussed on Twitter being news related [1]. Twitter can therefore be used as an information source in order to detect real world events. The content and metadata contained in the tweets can then be leveraged to describe the events and provide context and situational awareness. Applications of event detection and summarisation on Twitter have included the detection of disease outbreaks [2], natural disasters such as earthquakes [3] and reaction to sporting events [4].

Using the Twitter stream for event detection yields a variety of advantages. Normally in order to automatically detect real-world events a variety of official and media sources would have to be tracked. These are usually published with some lag time, and any system monitoring them programmatically would require customisation for each source since they are not formatted in any standard way. Twitter provides a real-time stream of information that can be accessed via a single API. In addition a rich variety of sources publish information to Twitter, since it is a forum both for the traditional media and for a newer brand of citizen journalists [5]. Tweets also contain metadata that can be mined for information, including location data, user-supplied hashtags and user profile information such as follower-friend relationships. The primary drawback of using Twitter is that it is an unstructured source that contains a great deal of noise along with its signal. Tweets can be inaccurate as a result of rumour, gossip or active manipulation via spamming.

In this paper we apply existing bio-surveillance algorithms, which are those used to detect outbreaks of illness, to detect candidate events from the Twitter stream. We employ customised filtering techniques to remove spurious events. We then extract the terms from the event tweets which best characterise the event and are most efficacious in retrieving related news. These terms, in the form of unigrams and bigrams, are used to filter and rank the most informative tweets for presentation to the user along with the most relevant news articles. Where the news articles cover the exact event being discussed on Twitter they act as direct confirmation and explanation for the event. Where a Twitter event has not yet been covered in the news media related background articles can still provide additional context.

Our techniques are evaluated using two case studies, both using a dataset of geo-located tweets from England and Wales collected in 2014. The primary case study is the detection of illness outbreak events. We then generalise our techniques to events strongly affecting Twitter sentiment, such as celebrity deaths and big sports matches. We evaluate our event detection using ground truth data in the form of health practitioner and news reports. The situational awareness techniques are evaluated by comparisons to existing term extraction methods and human-coded event explanations.

## Background

Much of the work on event detection using social media has focused on using topic detection methods to identify breaking news stories. Streaming document similarity measures [6], [7] and online incremental clustering [8] have been shown to be effective for this purpose. These methods have no concept of location, and focus purely on picking up distinct events being discussed in the general stream of Twitter data.

Other approaches have aimed to pick up more localised events. These have included searching for spatial clusters in tweets [9], leveraging the social network structure [10], analysing the patterns of communication activity [11] and identifying significant keywords by their spatial signature [12].

In the field of disease outbreak detection efforts have mostly focused on tracking levels of influenza by comparing them to the level of self-reported influenza on Twitter, in studies such as [13] and [14]. Existing disease outbreak detection algorithms have also been applied to Twitter data, for example in a case study [15] of a non-seasonal disease outbreak of Enterohemorrhagic Escherichia coli (EHEC) in Germany. They searched for tweets from Germany matching the keyword “EHEC”, and used the daily tweet counts as input to their epidemic detection algorithms. Using this methodology an alert for the EHEC outbreak was triggered before standard alerting procedures would have detected it. Our study uses a modified and generalised version of this event detection approach.

Diaz-Aviles et al. also attempted to summarize outbreak events by selecting the most relevant tweets, using a customized ranking algorithm. Other studies which have summarised events on Twitter by selecting the most relevant tweets include [4] and [16]. Analysis of using Twitter for situational awareness has been carried out in [17] and [18].

There have been fewer related works on linking or substantiating events detected from Twitter with traditional news media. One study [19] analysed various methods of contextualizing Twitter activities by linking them to news articles. The methods they examined included finding tweets with explicit URL links to news articles, using the content of tweets, hashtags and entity recognition. The best non-URL based strategy that they found was the comparison of named entities extracted from news articles using OpenCalais with the content of the tweets.

## Methods

### Problem definition

Our definition of a real-world event within the context of Twitter is taken from [8], with the exception that we have added a concept of event location. We are interested in only those events that attract discussion on Twitter, since all others would be invisible to our methods.

#### Definition 1.

(**Event**) An event is a real-world occurrence *e* with (1) an associated time period *T*_*e*_ and (2) a time-ordered stream of Twitter messages *M*_*e*_, of substantial volume, discussing the occurrence and published during time *T*_*e*_. The event has a location *L*_*e*_ where it took place, which may be specific or cover a large area, and the messages have a set of locations *L*_*M*1_,…, *L*_*Mn*_ which they were sent from.

From the above definition, when given a time-ordered stream of Twitter messages M, the event detection problem is therefore one of identifying the events *e*_1_,…, *e*_*n*_ that are present in this stream and their associated time periods *T*_*e*_ and messages *M*_*e*_. It is also valuable to identify the primary location or locations *L*_*Mi*_ that messages have originated from, and if possible the event location *L*_*e*_. The situational awareness problem is one of taking the time period *T*_*e*_ and messages *M*_*e*_ and producing an understandable summary of the event and its context.

### Overview

Our approach to the event detection problem incorporates location by detecting deviations from baseline levels of tweet activity in specific geographical areas. This allows us to track the location of messages relating to events, and in some cases determine the event location itself.

In this paper we focus on two distinct types of event:

- Outbreaks of symptoms of illness, such as coughing or itching.
- Events causing strong emotional reactions, such as happiness or sadness.

Initially the system was designed with disease outbreak detection as the primary use case; this led to a system design focused around keywords and aliases for their keywords, since a limited range of illness symptoms characterises most common diseases and the vocabulary used to describe these symptoms is also relatively limited. After several iterations of this approach we noted that it could be viable as a general event detection and situational awareness method, so we added another type of event to determine the feasibility of the general approach. We chose events causing strong emotions as a contrasting and less specific event type, with the intention of picking up a variety of localisable events such as important football matches and rock concerts.

For each type of event we define a list of keywords, each describing a particular sub-type of that event. For example when looking at illness each keyword relates to a particular symptom, such as ‘coughing’ or ‘vomiting’. When looking at events that cause emotional reactions each keyword relates to a particular emotion. Each of these sub-type keywords is then expanded with a list of aliases, synonyms and related terms to form a keyword group. For more details on how we identified the relevant keywords and synonyms see the sections below.

We track the number of tweets mentioning each keyword (consolidating all that lie in the same keyword group), in each of the geographical areas and use bio-surveillance algorithms to detect spikes in activity. Each spike is treated as a potential event, and we use various criteria to single out those with a high probability of being actual events as defined above, i.e. those that are caused by discussion of real-world occurrences on Twitter.

Our situational awareness approach is based on identifying terms from the event tweets which characterise the events and using them to retrieve relevant news articles and identify the most informative tweets. The news search uses metrics based on cosine similarity to ensure that searches return related groups of articles.

### Architecture

**Fig. 1:**
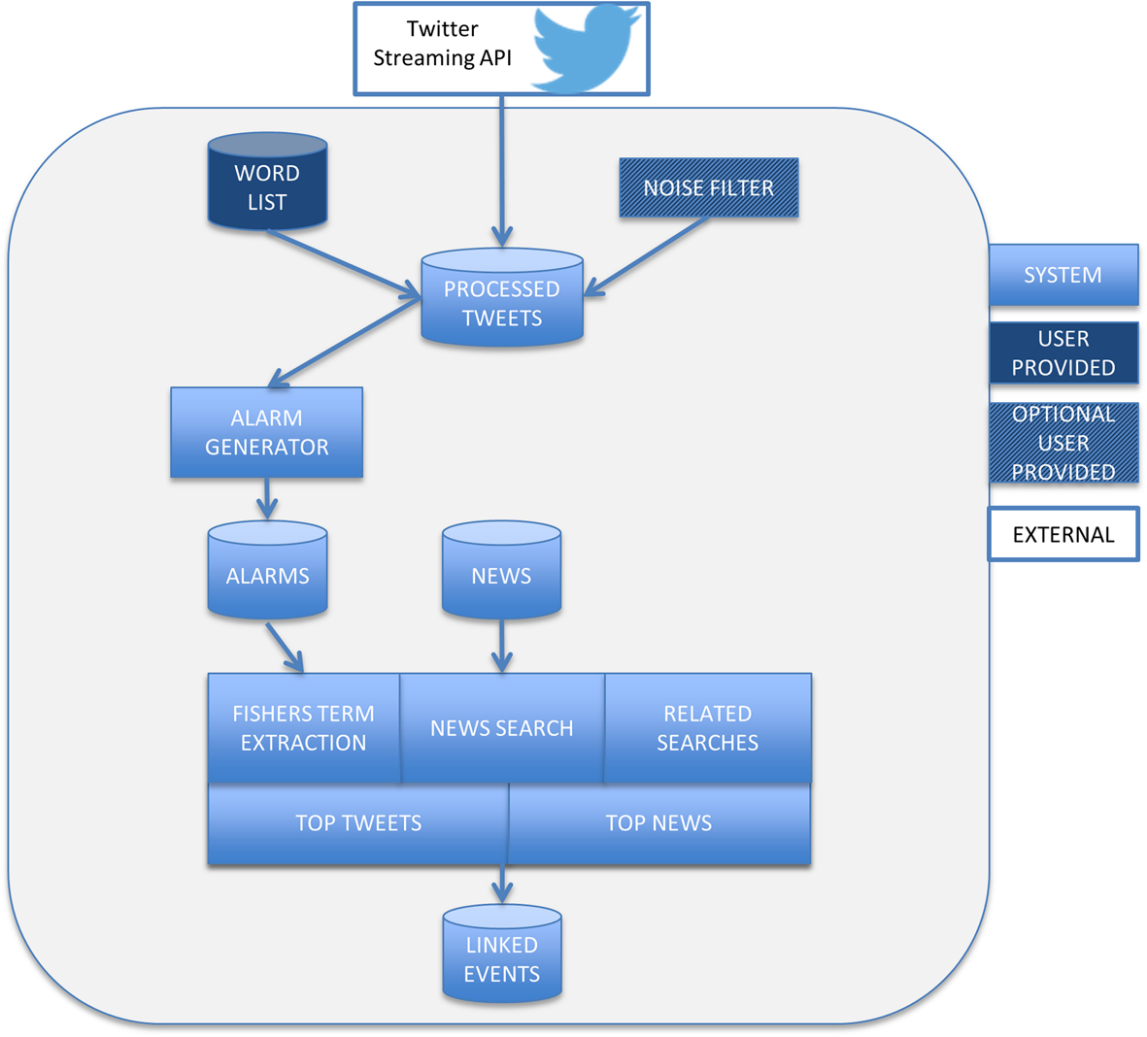
Event Detection and Situational Awareness architecture: To apply to a new example a user needs to provide a keyword group list and optionally a noise filter to remove tweets that do not strictly match the criteria of interest

The general approach can be described by the architecture in Fig. 1. Every new event type requires a list of keywords and their associated aliases. Optionally a specific data pre-processing step can be included for the event type. For example in the health symptom case we employ a machine learning classifier to remove noise (those tweets not actually concerning health). These are the only two aspects of the design that need to be altered to provide event detection and situational awareness to a new problem domain.

### Event types

We now go into a more detailed explanation of our event types and how we formulated the keywords and associated aliases. Each keyword group consists of a primary keyword which is used to identify the group, e.g. vomit, and a number of aliases that expand the group, e.g. throwing up, being sick, etc. see Tables 2 and 3 for the full list of keyword groups.

#### Illness symptoms

To build up a list of symptoms and related keywords we searched Freebase for /medicine/symptom. Each of these symptoms is defined as a keyword. They are returned with a list of aliases that are then associated with that keyword. This returned around 2000 symptoms. In order to filter down to a more manageable number we next filtered these symptoms by their frequency in the Twitter data; any symptoms not appearing frequently in this data would not produce enough activity to generate events for analysis. All symptoms with fewer than 10 mentions in the Twitter data were removed from the candidate list. This excluded a large proportion of symptoms, reducing the set to around 200.

We further limited the set by removing symptoms not related to infectious diseases. We also added primary keywords and aliases for some common conditions such as hayfever and flu. This step resulted in a reduction to the 46 symptoms which formed our search. The average number of aliases per primary keyword was 3.8.

#### Emotion states

For a list of emotion states and associated keywords we used the work of Shaver et al. They conducted research [20] to determine which sets of words were linked to emotions and how these cluster together. We took the six basic emotions identified in the work as primary keywords: *love, joy, surprise, sadness, anger and fear*. Shaver’s work associated each of these with a list of terms to form a tree. We took the terms from lower leaves on the tree for each emotion as our alias sets (see Table 1 for examples). The average number of aliases per primary emotion keyword was 7.3. The only alteration we made was that after some initial analysis we discovered that the term “happy” from the “joy” category was a very strong signal of special events such as Valentine’s Day, Mother’s Day and Easter. It was also very often used on a daily basis due to people offering birthday greetings. We therefore separated “happy” into its own category separate from “joy”.

**Table 1:** Selected emotion keyword groups and some of their aliases

Keyword	Aliases
Surprise	Amazed, astonished, surprised...
Sadness	Depressed, unhappy, crying...
Joy	Glad, delighted, pleased...

In addition we employed SentiStrength [21], a sentiment analysis tool, to classify our tweets into positive and negative emotional sentiments. We took those tweets classified as being very positive and very negative as additional categories.

### Data collection

Using Twitter’s live streaming API we collected geo-tagged tweets between 11th February 2014 and 11th October 2014. Tweets were collected from within a geographical bounding box containing England and Wales. Retweets were excluded due to our focus on tweets as primary reports or reactions to events. This resulted in a data-set of 95,852,214 tweets from 1,230,015 users. 1.6 % of users geo-tag their tweets [22], so our data is a limited sample of the total tweet volume from England and Wales during this period. We chose to use only geo-tagged tweets since they contain metadata giving an accurate location for the user. This allows us to locate each tweet within our geographical model. In total we found 240,928 matches for our symptom keywords in the set of tweets classified as health-related, and 20,570,753 matches for our emotion keywords. See Tables 2 and 3 for details.

**Table 2:** Tweets matching each symptom keyword group

Symptom	Number of tweets
Headache	42947
Vomit	30429
Hayfever	24175
Sore throat	21744
Pain	15142
Malaise	12354
Flu	10913
Cough	9589
Tonsillitis	7283
Common cold	6768
Infection	5955
Abdominal pain	5582
Sneeze	5131
Asthma	4457
Shortness of breath	4037
Earache	2990
Nasal congestion	2930
Tremor	2727
Itch	2410
Anxiety	2250
Fever	2198
Nosebleed	1971
Faint	1944
Skin rash	1633
Cramp	1444
Diarrhea	1365
Chest pain	1293
Swollen gland	1138
Conjunctivitis	941
Stinging sensation	891
Bleeding	854
Chickenpox	835
Runny nose	785
Swelling	692
Meningitis	641
Pneumonia	622
Seizure	413
Constipation	389
Palpitation	360
Norovirus	239
Neck pain	203
Scarlet fever	142
Dehydration	68
Dysentery	28
Tearing	16
Dry mouth	10

**Table 3:** Tweets matching each emotion keyword group

Emotion	Number of tweets
Very negative	5716797
Love	4943706
Very positive	3823994
Joy	2129279
Happy	1613447
Anger	1228890
Sadness	562193
Fear	395770
Surprise	156677

### Location assignment

Our methodology relies on the collection of baseline levels of tweet activity in an area, so that alarms can be triggered when this activity increases. We therefore amalgamated the fine-grained location information from the geo-coded tweets by assigning them to broader geographical areas. We used a data driven approach to generate the geographical areas rather than using administrative areas such as towns or counties. This technique allowed us to select only those areas with a minimum level of tweet activity, and also did not require any additional map data. It would therefore be be reusable for any region or country with a sufficient level of Twitter usage.

We began by viewing a sample of the collected tweets as geo-spatial points. Viewed on a map these clearly clustered in the densely populated areas of England and Wales. We therefore decided to use a clustering algorithm on these points in order to separate out areas for study. We employed the Density-Based Spatial Clustering of Applications with Noise (DBSCAN) algorithm [23] for clustering, as this does not require a priori knowledge of the number of clusters in the data. The features provided to DBSCAN were the latitudes and longitudes of the tweets.

**Fig. 2:**
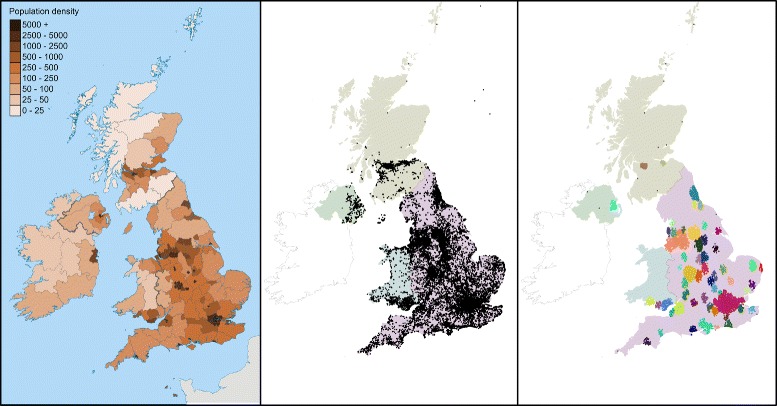
UK population density (*left*) compared to a sample of geo-located tweets (*centre*) and the clusters found (*right*). Note that only clusters located in England and Wales were used in this study. Contains Ordnance Survey data c Crown copyright and database right, CC BY-SA 3.0, https://commons.wikimedia.org/w/index. php?curid=26070175

The clusters produced by the algorithm matched the most populated areas, corresponding to the largest cities in the UK as shown in Fig. 2. They also separated most cities into distinct clusters (a notable exception being the conglomeration of Liverpool and Manchester). In total 39 clusters were created for England and Wales and each was given an ID and a label. We then created a convex hull around each cluster, providing a polygon that can be used to check whether a point is in the cluster or outside it. Points outside all of the clusters were assigned to a special ‘noise’ cluster, and not included in the analysis. Overall 80 % of tweets were assigned to specific clusters and the remainder to noise, giving us good coverage of geo-tagged tweets using our cluster areas.

### Tweet processing

As tweets are received by our system they are processed and assigned to the symptom and emotion state classes if they contain one of the relevant keywords. They are assigned a location by checking whether they fall into one of our cluster areas.

For the illness symptoms we introduce a noise removal stage at this point. It is particularly relevant for this class of events because there are many fewer tweets relating to illness than showing emotion states. This means that the signal is more easily blocked out by random noise. To remove noise we construct a machine learning classifier with the aim of removing tweets containing alternative word usages or general illness discussion rather than reporting of illness events. The classifier therefore classifies tweets into those that are self-reports of illness and those that are not. The classifier we use is a linear SVM trained on a semi-supervised cascading training set, created on the principles described in Sadilek et al. [24]. Our classifier uses the LibSVM [25] library, and was initially trained on 4600 manually classified tweets. It achieves a classification accuracy of 96.1 % on a held out test set of 920 manually classified tweets.

The number of tweets assigned to each class in each area are then saved on a daily basis. These counts are first normalised to take account of Twitter’s daily effect pattern, which shows more tweeting on weekends than weekdays. Event detection is run daily since we are attempting to pick up temporally coarse-grained events. Disease outbreaks take weeks to develop, and events that shift public sentiment or emotion will generally take hours or days to unfold.

### Detecting events

Our event detection methodology leverages considerable existing syndromic surveillance research by using an algorithm designed and developed by the Centers for Disease Control and Prevention (CDC), the Early Aberration Reporting System (EARS) [26].

#### Definition 2.

(**Alarm**) An *alarm* is an alert produced by the first stage of our event detection system. The alarm has an associated keyword group and location. It also has a start and end date, and associated tweet counts for each date within this period. When certain criteria are met an alarm is deemed to be an event.

We employ the C2 and C3 variants of EARS. These algorithms operate on a time series of count data, which in our case is a count of daily symptomatic tweet activity. The C2 algorithm uses a sliding seven day baseline, and signals an alarm for a time *t* when the difference between the actual count at *t* and the moving average at *t* exceeds 3 standard deviations. The C3 algorithm is based on C2, and in effect triggers when there have been multiple C2 alarms over the previous 3 days.

These C2 and C3 candidate alarms are then grouped together so that alarms for the same keyword group and area on consecutive days are treated as a single alarm. An alarm is therefore made up of one or more days, each with an observed count of tweets. An alarm ends when the C2 and C3 algorithms no longer signal an outbreak occurring.

Some of our Twitter count time series data is zero-skewed and non-normal, since the number of geo-tagged users reporting illness can be low. The number of standard deviations from the mean used in the C2 and C3 algorithms can be an unreliable measure of central tendency in those circumstances. Hence to determine how far above general baseline activity an observed count is we employ the median of the series to date and its Median Absolute Deviation (MAD) to produce a new metric of alarm severity. Here the series is defined as all of the previous observed counts for the keyword group and location in question. The number of Median Absolute Deviations from the median, *μ*, gives a comparable figure across alarms as to how sharp a rise has been over expected levels. This figure is produced from the following equation:

1 $$ \mu = (observation-median)/MAD  $$

We then find the highest metric for an alarm, *μ*_*max*_, by finding the highest value of *μ* within the observations making up the alarm.

2 $$ \mu_{max} = \operatorname*{arg\,max}_{\mu} (observations in alarm)  $$

The *μ*_*max*_ is the primary statistic which we use to determine which events are real and which have just been generated by random noise.

Another statistic which we employ in order to filter out noise is the tweet-user ratio. This is the ratio of tweets in an event to that of distinct users involved in an event. A high value of this statistic would imply that some users have tweeted a large number of times across a short time period, which is an indication that they may be spammers and that the alarm is spurious.

In summary, we use the output from EARS to produce alarms. We filter the alarms to a set of high likelihood events by using the *μ*_*max*_ and tweet-user ratio parameters. From this point we refer to those alarms that are high-likelihood as events, according to our earlier event definition. The alarms have an associated stream of Twitter messages and a location given by the node which they occur in. The following situational awareness results show that the Twitter messages in these alarms discuss real-world occurrences, therefore fulfilling all of our definition.

### Situational awareness

Once an event has been identified our next objective is to automatically provide additional context for it, which may provide an explanation of the underlying cause. A human interpreter could achieve this by reading all of the tweets and synthesizing them into a textual explanation, which might be some text such as “People reacting to the death of Robin Williams”. We do this in two main ways: by providing the most representative tweets from those that triggered the alarm, and by linking to relevant news articles. The steps involved in the Terms, News and Tweets (TNT) Event Summarisation process are detailed in Algorithm 1. The steps and terminology are then explained in more detail.



① The first step is to retrieve the relevant tweets from the processed tweet and alarm databases. Tweets are fetched for both the alarm gist and from a historical baseline. ③ We discard those events with fewer than 30 tweets as we found that they did not contain sufficient data to produce good summarisation results.

#### Definition 3.

(**Gist**) The gist consists of the tweets for the time period of the event which match the event’s keyword group and area.

#### Definition 4.

(**Baseline**) The baseline consists of the tweets for the same keyword group and area as an event from the 28 days prior to that event.

⑤ The next task is to find unigrams and bigrams that are more prevalent in the gist than in the baseline. These are likely to come from tweets discussing the event and will thus be characteristic of the event. We first extract the most common unigrams and bigrams from both sets of tweets, after removal of stopwords. Our list of stopwords includes a standard list, plus the 200 most frequent words from our tweet database. We select all non-stopwords that appear in at least 5 % of the tweets.

⑦ We then do a Fisher’s Exact Test to determine which of the common unigrams and bigrams in the gist appear significantly more frequently (*α*<0.05) here than in the baseline set. Our candidate terms are the top two most significant unigrams and bigrams. We select the top two as this was found to give the best results on our test examples. To this set we append the primary keyword that triggered the alarm.

⑨ For this research Google was used as the news database. Using the candidate terms we perform a search on Google for documents published in the United Kingdom during the time period of the alarm. Due to Google’s Terms of Service this step was performed manually. A fully automated system would replace this step with a search of a news database, which could be created by pulling down news articles from RSS feeds of major content providers.

⑩ We take the first 10 documents retrieved for each search term, remove stopwords and apply stemming using a Lancaster stemmer. We then convert each document into a Term Frequency/Inverse Document Frequency (TF/IDF) vector. In order to determine whether the search term has retrieved a coherent set of related documents we define a metric based on cosine similarity, the Pairwise Cosine Similarity Score (PCSS):

- The **Pairwise Cosine Similarity Score** of a group of TF/IDF vectors is calculated by taking the cosine similarity between each pair of vectors and adding them to a set. The standard deviation of this set is subtracted from its mean to form a score.

The PCSS rewards articles which are similar and penalises any variance across those article similarities. This reduces the effect of some articles being strongly related in the document set and others being highly unrelated. Any term which retrieves a set of documents with a score below a threshold value is not considered further.

It is possible for a search term to hit on a coherent set of documents purely by chance, perhaps by finding news articles related to another event in a different part of the world. In order to guard against this we institute another check to ensure that the set of documents returned from a search term is sufficiently closely related to the set returned from at least one other search term.

⑫ In order to perform this check we compare the titles of the articles returned from the two different searches using a similar process to our earlier document comparison. We found it more effective to compare titles than whole documents, since sets of documents with similar topics can contain similar language even for fairly unrelated search terms. For example the terms “ebola” and “flu” will both return health-related documents containing similar language, but we would not wish to say that these search terms are related. To convert the titles to TF/IDF vectors we remove stopwords but do not apply stemming. Since the titles are so short we include all unigrams, bigrams and trigrams in the vector representation. We then compute a PCSS between the two document sets, pairing each document in the first set with each in the second and vice versa. ⑬ A search term must be related to at least one other term for it to be used going forward.

⑭ Once TNT has identified good search terms we then return the news articles fetched using those terms. ⑯ In order to rank the top news articles for a search we take the mean TF/IDF vector of the articles. and then rank the articles by cosine similarity to this mean vector. We return the top ranked articles from each search term.

⑰ To select the summary tweets for an event we firstly determine the set of tweets to consider and then choose the most relevant tweets within that set. The set of tweets can either be:

- 1) All tweets in the gist.
- 2) The gist tweets containing one of the extracted terms.
- 3) The gist tweets containing one of the ‘good’ search terms (as determined by the TNT algorithm).

1) is always available and is labelled the *Gist Top Tweets* (GTT). If the TNT algorithm has found terms that are significantly different in frequency from the baseline then set 2) is available for use and if terms from that set have good news matches then set 3) can be used. The *Summary Top Tweets* (STT) are from set 3) if it exists and fallback to set 2) if the good news match terms are not available. If no terms were found to be significantly different from the baseline then only the GTT is available.

In order to choose the top tweets we rank them by their cosine similarity to the mean TF/IDF vector of all tweets in the set, an approach similar to that of [4]. This attempts to finds tweets which capture and summarise the aggregate information of all of those in the set. The top five tweets ranked by this measure are returned.

## Results and discussion

### Candidate event selection

Over the course of the study the bio-surveillance algorithms generated 820 disease-related alarms and 2021 emotion-related alarms. A brief survey of these revealed that many were false alarms generated by random fluctuations in the noisy social media data. In order to separate out alarms that could be labelled as events with high confidence we conducted the following analysis.

Firstly we compiled an initial set of 13 focus example alarms. These were taken from events that the authors knew had happened in the evaluation time period and from those alarms in our dataset with low and high values of *μ*_*max*_.

**Fig. 3:**
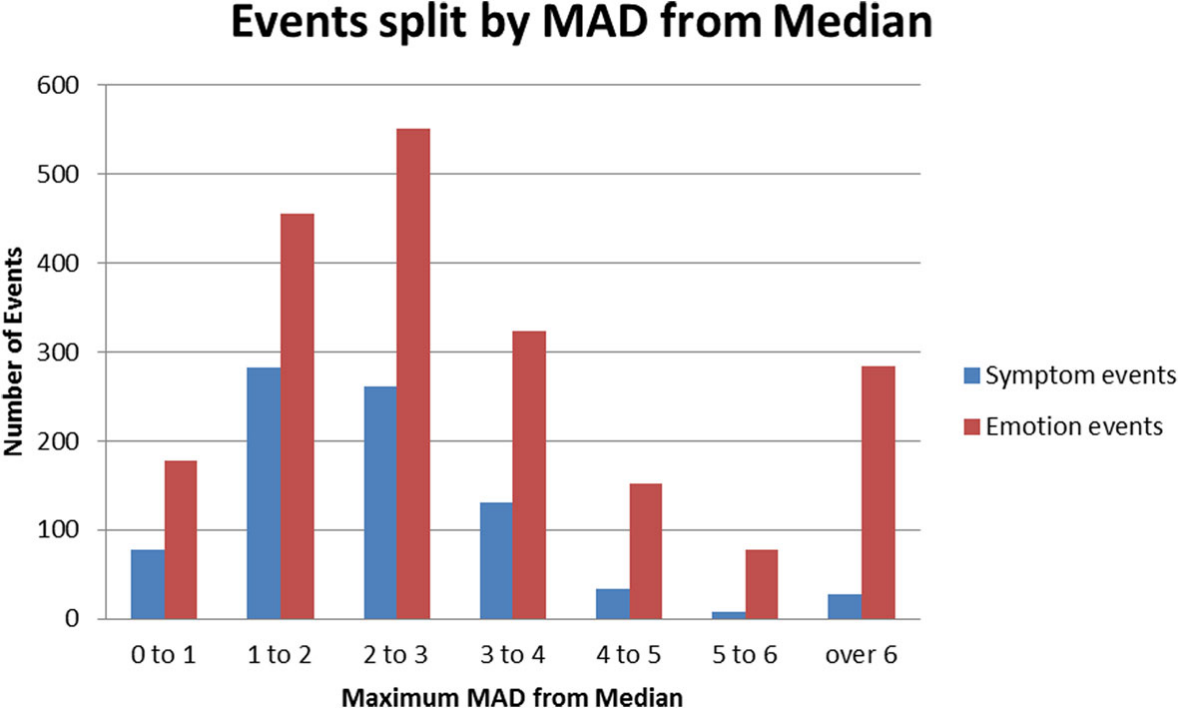
Alarms detected with differing values of *μ*_*max*_

The most important threshold parameter in the context of the event detection is the *μ*_*max*_ figure which measures the deviation of the alarm counts from the median level. Examining the distribution of the number of alarms for each value of *μ*_*max*_ revealed that it started to tail off sharply at *μ*_*max*_≥5. The distribution of alarms for each value of *μ*_*max*_ is shown in Fig. 3. We therefore took this as a value to segment additional test examples, drawing ten more at random with a *μ*_*max*_ less than 5 and ten with a *μ*_*max*_ greater than or equal to 5. The resulting evaluation set of 33 candidate events is shown in Table 4. The event ID used to refer to the events is composed of the first two letters of the event keyword followed by a 1–2 letter area code. The final part of the ID is the day and month of the event start date.

**Table 4:** Evaluation set of events

ID	Event	*μ*_*max*_	Keyword	Node	ID	Event	*μ*_*max*_	Keyword	Node
SAL-11-08	YES	20	Sadness	London	HFB-10-04	YES	5	Hayfever	Birmingham
HFM-01-06	YES	19	Hayfever	Manchester	VOL-20-04	YES	5	Vomit	London
SAL-07-04	YES	14	Sadness	London	SAC-05-05	YES	5	Sadness	Cardiff
FEL-18-07	YES	13	Fear	London	HFL-04-07	NO	5	Hayfever	London
ASL-02-04	YES	12	Asthma	London	FLB-23-09	NO^a^	5	Flu	Birmingham
FLP-06-10	YES	11	Flu	Portsmouth	VPBR-10-05	YES	4	VeryPos	Bristol
HAM-02-04	YES	9	Happy	Manchester	FRL-30-05	YES	4	Fever	London
HAM-18-04	YES	9	Happy	Manchester	FLM-19-09	YES	4	Flu	Manchester
SAL-08-07	YES	8	Sadness	London	VOL-22-02	NO	3	Vomit	London
HALE-01-08	YES	8	Happy	Leeds	HFB-29-04	NO	3	Hayfever	Birmingham
HFL-14-05	YES	7	Hayfever	Leeds	JONO-23-02	YES	2	Joy	Norwich
SUN-29-08	YES	7	Surprise	Newcastle	HEM-06-03	NO	2	Headache	Manchester
ITL-08-06	YES	6	Itch	London	SUC-23-05	NO	2	Surprise	Cardiff
SAB-09-06	YES	6	Sadness	Birmingham	SUL-16-08	NO	1	Surprise	London
HABE-01-03	YES	5	Happy	Bridgend	FEBR-17-04	NO	0	Fear	Bristol
SAL-21-03	YES	5	Sadness	London	STL-26-08	NO	0	Sore Throat	London
HFC-09-04	YES	5	Hayfever	Cardiff					

Shows whether events were externally verifiable and their *μ*_*max*_ value

^a^Note: this event not confirmed by the GP in hours report of that week. However, the following week showed an increase and it is possible that social media detected increased Influenza activity before this was confirmed by GP visits

### Event detection evaluation

It is difficult to provide a completely automated evaluation procedure for detecting previously unknown events. Diaz et al. used the time to detection on a known outbreak as their evaluation criterion [15]. In our case we do not know a priori that these are genuine outbreaks or events. Hence we need to make an assessment of the alarms produced to see what they refer to and if there is a way of externally verifying that they are genuine events. For all 33 of the selected alarms the authors read the tweets and determined whether they described a real world event. The coders found 26 YES answers, 5 NO answers and 2 DISAGREED answers, producing a 94 % agreement. Where an event was present they wrote a short summary.

For external verification of events two different methods were used, depending on whether the event was symptom-related or emotion-based. For symptom related events the activity spike was checked against official sources for the same time period. The General Practitioner (GP) in hours bulletin for England and Wales [27] was used and an event was deemed verified if the symptom exhibited an increasing trend for that period. This detail is noted in the summary document produced by Public Health England for that reporting period. Emotion-based events were verified by checking if there were any articles (via Web search) that could corroborate the cause of the event (as given by the summary).

We manually investigated all examples from the initial focus set and found initial parameters for the score functions in our algorithms that worked reasonably well. These provided possible ranges of values which were evaluated more systematically over the entire alarm set. For event detection we evaluated which alarms were flagged as events by the system for each parameter value against whether those events were externally verifiable. The final evaluation for all algorithms contains all 33 of the alarms in both sets, not just the twenty expanded ‘test’ examples.

To determine if an alarm is an event that we should be concerned about we consider two properties of the alarm. The first is the tweet-user ratio. From exploratory testing we found a value of 1.5 separated our spam and genuine alarms very well, leaving only a small number of alarms with large tweet sets and some spam. The spam detection problem should be straightforward and will be addressed more completely in future work.

The second figure which gives the strength of the activity above the usual baseline is the *μ*_*max*_ figure. This is the essence of the modified EARS algorithm and the value of this figure should generally separate events from non-events.

The criterion for selecting the best threshold for *μ*_*max*_ is context dependent. We have used the balanced measure for this scenario as that is a fair representation of both precision and recall. For each threshold value of *μ*_*max*_ tested the classification success and error types are:

- **True positive**: instances at or above the threshold that are verified events
- **False positive**: instances at or above the threshold that are not verified events
- **True negative**: instances below the threshold that are not verified events
- **False negative**: instances below the threshold that are verified events

**Fig. 4:**
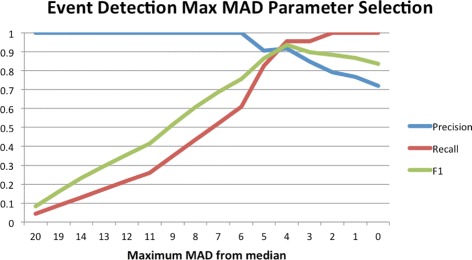
*μ*_*max*_ event detection parameter selection

The precision, recall and F1 values for all the tested values of *μ*_*max*_ are displayed in Fig. 4. All figures were calculated with reference to the set of 33 example events discussed above. The maximum F1 value, 0.9362, is observed at *μ*_*max*_≥4, so this is a well balanced threshold and the recommended parameter. Those seeking higher confidence events (willing to accept that some events may be missed) could use a value of 6 for this parameter which yields a precision of 1. The maximum observed recall value is at the minimum parameter value and is not very informative. Essentially it says that everything is an event and hence does not produce any false negatives.

In summary the event detection mechanism based on the EARS C2 and C3 algorithms with the addition of the *μ*_*max*_ and tweet-user ratio was found to perform well at detecting events that could be externally verified as genuine. The recommended *μ*_*max*_ parameter (4) produced a good balance of precision and recall in our sample set. It must be noted however that we cannot gain a true picture of the overall recall of the system, since we have no way of analysing the number of genuine events that were not picked up.

### Situational awareness evaluation

Both situational awareness components were evaluated. Firstly the news linkage was tested to see whether relevant news was retrieved for the sample events. As part of this analysis we compared our method of extracting informative search terms (the TNT algorithm) with a comparable automated technique. Secondly the tweet ranking was validated to determine whether highly ranked tweets effectively summarised the events.

#### Comparative news linkage evaluation

The news linkage component works by selecting good search terms for articles based on the TNT algorithm. Within this there is a term extraction step to generate search terms, and then a filtering step using PCSS to remove terms which retrieve unrelated sets of articles. We iterate over different threshold values for the PCSS score to find the optimum, using an F0.5 measure as the evaluation criterion. F0.5 was selected because precision was judged to be more important than recall in this setting. As a further evaluation we compare the results of replacing our term extraction algorithm with Latent Dirichlet Allocation (LDA). LDA is a popular topic modelling technique that extracts sets of terms characterising each topic in a group of documents. The success and error types used to compute the F0.5 measure are:

- **True positive**: relevant news returned for newsworthy event
- **False positive**: news returned for an event with no genuine news
- **True negative**: no news returned for an event with no genuine news
- **False negative**: no news returned for newsworthy event

**Fig. 5:**
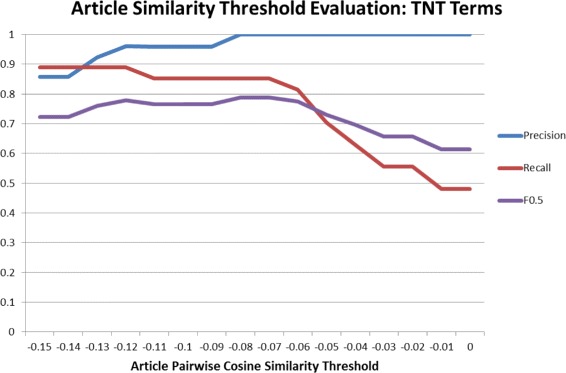
News linkage accuracy from Terms, News, Tweets terms

**Fig. 6:**
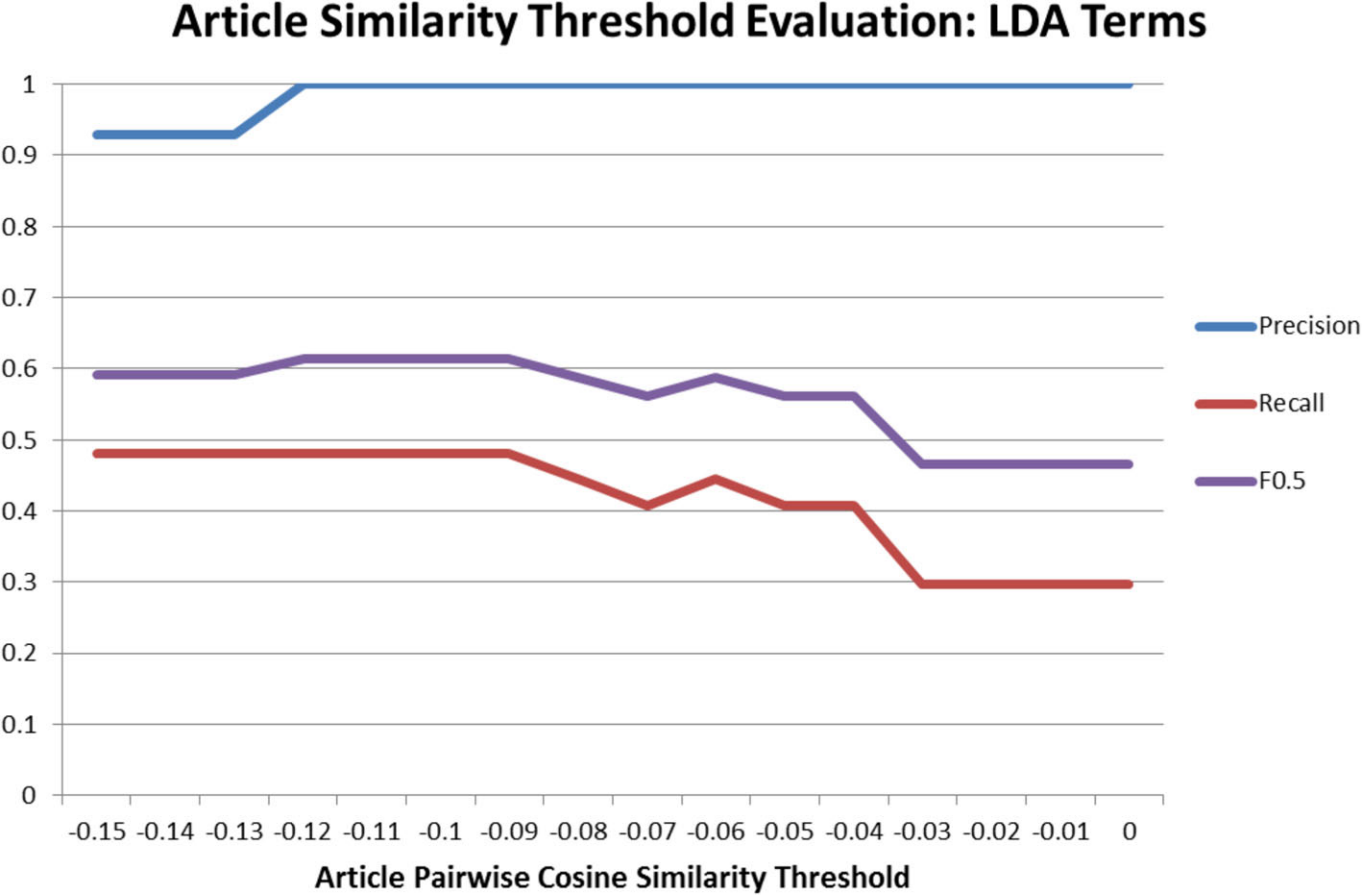
News linkage accuracy from Latent Dirichlet Allocation terms

The evaluation is presented in Figs. 5 and 6 as well as the different levels of article PCSS that were iterated over to find the maximum F0.5 value in a step-wise procedure. It is clear from these images that the TNT algorithm has a higher F0.5 at all tested values of the article PCSS, due to its higher recall. The outcome of the parameter selection process was that a PCSS threshold of −0.08 produced the best results. Using this value the F0.5 was 0.79, showing that our system was successful in retrieving relevant news for the sample events.

Selecting top ranked relevant news articles is one part of our situational awareness contribution. The second is the selection of tweets that provide a representative summary of an event.

#### Top ranked tweets evaluation

We have employed two evaluations for the tweet ranking exercise: comparison to human-coded event explanation and comparison between GTT and STT. The human-coded event explanations were created by two of the authors after reading through all of the tweets linked to each event. There were 26 alarms that had an identifiable cause. The tweet ranking match (to human-coded event assessment) performance is presented in Table 5. The tweets were considered a full match if a human summary of the 5 top ranked tweets would match the human-coded event explanation for the whole set of tweets.

**Table 5:** STT tweet ranking evaluation

Match	Count
Full	21
Partial	2
No	3

The STT tweet summary fully matched the human-coded event summarisation in 21 cases. This yields a full match fraction of 0.81

The partial matches were: FRL-30-05 (Fever: London, May) and FLP-06-10 (Flu: Birmingham, October). These events had more than one explanatory cause. Currently our algorithms work best in the single event case. The three cases that did not match were: JONO-23-02 (Joy: Norwich, February), STL-26-08 (Sore throat: London, August) and SUN-29-08 (Surprise, Newcastle, August). The coders disagreed as to whether STL-26-08 was actually an event. The remaining two examples were not summarised well by the significant tweets as they both exhibited high disparity in terms used to describe a contextually related event and SUN-29-08 also included a number of spam tweets that distorted the results of TNT.

The second evaluation for the tweet ranking exercise was a comparison between the GTT and the STT. A qualitative assessment of the tweets led to the conclusion that STT tweets were better in 11 out of 33 cases and there was no significant difference between the two for 21 cases out of 33. In one case, FLP-06-10, the GTT included a mention of “flu jab” (one of the manually selected terms) which the STT did not include. Hence the STT provided an improvement over ranking based off the alarm tweets in one out of three instances.

### Notable examples discussion

We now discuss four example events that highlight the strengths and limitations of our approach. These examples are listed in Table 6.

**Table 6:** Example cases and the terms extracted for them

ID	TNT terms	LDA terms
JONO-23-02	Joy, enjoy	Enjoy, glad, loss
ASL-02-04	Asthma, air pollution, smog, pollution	Asthma, smog, pollution, attack air
VOL-20-04	Vomit, chocolate, easter	Chocolate, eaten, easter, vomit, headache
SAL-11-08	Sadness, robin williams, sad news, robin, williams	Sad, robin, williams, rip, riprobinwilliams

The first example case is JONO-23-02. From a reading of the tweets there were definitely some relating to a single event: Norwich City Football Club beating Tottenham Hotspur Football Club 1−0 in a football match. Both TNT and LDA term extraction failed to find terms representative of this event. This was due to the disparity of the language used; the following example tweets should help elucidate this point:

- *#canarycall absolutely delighted with the win :) good performance, good result*
- *#yellows almost didn’t go today glad i did*
- *so glad i chose to come today!#ncfc*

It is difficult for a term-based solution to find any common thread here. Finding the cause of this event would require contextual knowledge of football matches, team names and commonly employed aliases. The news linkage algorithm did initially find a news story for the term “joy” on this date. *The British Prime Minister “let out a little cry of joy” over David Bowie Scottish independence comments* (Telegraph, Feb 24, 2014). The articles returned all concerned this story and were found to be closely related, but were dropped from the news linkage because they did not match those returned from the other search terms. This highlights the benefits of searching with multiple terms and ensuring that the results are related.

The second example is ASL-02-04. This event was due to increased levels of air pollution observed in London at the beginning of April, caused by a Saharan dust cloud. This event had a *μ*_*max*_ of 12 indicating a significant increase in baseline activity for the alert period. It was well summarised by all aspects of our situational awareness algorithm. The top ranked tweets provided by our summary method (STT) produced tweets more representative of the event than those from all tweets in the gist. This is demonstrated by the top tweet selected by both:

- STT top tweet: *i can’t breathe #asthma #smog*
- GTT top tweet: *my asthma is literally so bad*

Here selecting the top tweets from the filtered event set captures tweets representative of the event as opposed to the baseline illness activity. The news linkage for this example worked well, with all five of the top selected articles being representative of the event. The top article, *“Air pollution reaches high levels in parts of England - BBC”*, gives the cause of the event in the first few lines: *“People with health problems have been warned to take particular care because of the pollution - a mix of local emissions and dust from the Sahara.”*

The third case is VOL-20-04. Reading the tweets makes it clear that this one day event is caused by people feeling sick after eating too much chocolate on Easter Sunday. In this case the TNT summary and all tweet ranking return similar tweets as there is little baseline activity and that baseline activity is not strongly related. The top tweets from both sets therefore both produce good summaries:

- STT top tweet: *seriously i feel sick having all this chocolate*
- GTT top tweet: *eaten too much chocolate feel sick*

While the top ranked tweets are similar the event tweet filtering does remove baseline tweets referring to general illness. No good news searches were found in this case. This event may be valid in the context of social media but it is not newsworthy.

The fourth example is SAL-11-08 which is the UK Twitter reaction to the death of Robin Williams. These tweets from the sadness keyword group exhibit both the highest *μ*_*max*_ (20) and the highest overall tweet count for any single event (4472). The prominence of celebrity deaths within our detected events mirrors earlier findings [6]. As with all of our high *μ*_*max*_ events the TNT tweet ranking and news linkage work well. The top news article returned is an article reporting the death of Mr. Williams: *“Robin Williams dies aged 63 in suspected suicide”* (Telegraph, August 12, 2014). The top five ranked tweets by TNT tweet filtering are better than those ranked on all tweets as they remove baseline general sadness tweets from the ranking:

- STT top tweet: *rip robin williams. sad day*
- GTT top tweet: *yep, very sad*

## Conclusions

We have presented techniques for event detection and situational awareness based on Twitter data. We have shown that they are robust and generalisable to different event classes. New event classes could be added to this system simply by producing a list of keywords of interest and an optional noise filter. Our event detection is based on the EARS bio-surveillance algorithm with a novel filtering mechanism. The maximum Median Absolute Deviations from the median provides a robust statistic for determining the strength of relative spikes in count-based time series. As it is based on the median, this measure handles cases where data is non-normal as was the case for some of our symptom based geo-tagged tweets. The event detection approach achieved an F1 score of 0.9362 on our event examples.

By filtering to words that are significantly different (*α*<0.05) in frequency from baseline levels we have extracted terms to search news sources for related articles. Where good news matches are found these revise our event term list. We have created two novel algorithms that provide additional situational awareness about an event from these event terms. The baseline tweet activity thus provides valuable context in allowing the character of the detected event to be discerned.

Firstly, we rank the filtered set of news articles to produce the top five representative articles. The news linkage, weighted towards precision, achieved an F0.5 score of 0.79 on our example set, with no false positives.

Secondly, we produce a top five ranked list of tweets that summarise an event. These ranked tweets are calculated from the tweet set, filtered by those that contain the extracted event terms. The top ranked tweets fully matched our human-coded event summaries in 21 out of 26 cases.

In future work we aim to improve our news linkage algorithm with a final step checking whether the articles returned are similar to the event tweets, using cosine similarity or other features such as entities identified in the news articles. Additional improvements to event detection would lie in improving spam detection and adding sentiment classification to our emotion example as a classifier. Collecting data over longer time periods would also allow us to look into using bio-surveillance algorithms which require seasonal baseline information.
